# Age-Stratified Indications and Complications of Girdlestone Hip Resection Arthroplasty: A Retrospective Cohort Study

**DOI:** 10.21203/rs.3.rs-10695380/v1

**Published:** 2026-09-14

**Authors:** Carolyn Henein, Mario Eusebio, Christopher Hamad, Jack Pearce, Timothy Liu, Nicole Truong, Mark M Herbert, Chinonyelum Ikeanyi, Lauren Hsue, Ava Jafarpour, David Chukwu, Mauricio Silva, Soroush Baghdadi

**Affiliations:** The University of Texas Medical Branch at Galveston; University of California, Los Angeles; University of California, Los Angeles; University of Vermont; University of California, Los Angeles; University of California, Los Angeles; University of California, Los Angeles; University of California, Los Angeles; University of California, Los Angeles; University of California, Los Angeles; University of California, Los Angeles; University of California, Los Angeles; University of California, Los Angeles

**Keywords:** Girdlestone resection arthroplasty, proximal femoral resection, cerebral palsy, periprosthetic joint infection, pediatric orthopaedics, hip salvage surgery

## Abstract

**Background:**

Girdlestone hip resection arthroplasty (GRA) is a salvage procedure reserved for recalcitrant periprosthetic infection, failed arthroplasties, as well as neuromuscular hip dislocations in children. Existing evidence derives largely from single-institution, age-restricted studies. Therefore, the purpose of this study was to report demographic, diagnostic, and complication profiles of GRA across the population in a large retrospective cohort.

**Methods:**

We conducted a retrospective cohort study using the TriNetX Research Network, a federated network of deidentified electronic health record data. Patients who underwent Girdlestone resection arthroplasty between October 2015 and June 2024 were identified and stratified into pediatric (≤ 18 years) and adult (> 18 years) cohorts. Demographics, the ten most prevalent International Classification of Diseases, Tenth Revision, Clinical Modification (ICD-10-CM) diagnoses per cohort, and postoperative outcomes, including return to the operating room, pain, opioid use, heterotopic ossification, decubitus ulcers, infection, and death, were assessed.

**Results:**

A total of 1,876 patients were identified: 87 pediatric (≤ 18 years) and 1,789 adult (> 18 years) patients. The leading pediatric diagnosis was cerebral palsy. The adult diagnostic profile was less homogeneous and included unclassified pain (4%) and other unclassified joint disorder (4%), followed by pressure ulcer, paraplegia/quadriplegia, and cerebral palsy (3% each). A diagnosis of cerebral palsy was present in both cohorts (28% pediatric vs 3% adult). Pain occurred at similar rates in both groups (57.5% pediatric vs 60.8% adult; P = 0.575). Opioid use (11.7%), return to the operating room (10.0%), decubitus ulcers (28.3%), surgical site infection (5.3%), pyogenic arthritis (23.6%), osteomyelitis (12.8%), sepsis (9.1%), and death (11.4%) occurred only in the adult cohort (all P ≤ 0.020). Heterotopic ossification occurred only in adults (1.1%; P = 1.000).

**Conclusions:**

Girdlestone resection arthroplasty was associated with distinct indications and outcomes across age groups. Pediatric patients underwent the procedure predominantly for neuromuscular hip disease, while adults carried a high burden of infection, reoperation, and mortality. These findings support age-specific risk counseling rather than a single composite risk estimate.

**Type of study/level of evidence:**

Retrospective comparative study; Level III.

## Introduction

Girdlestone resection arthroplasty (GRA) of the hip was originally described as a salvage operation for tuberculosis of the hip.^1^ Although total hip arthroplasty and staged reconstruction have largely replaced GRA as definitive treatments, the procedure persists in contemporary practice as a salvage option when hip reconstruction is no longer feasible or has failed.^2–4^

Today, GRA is performed across a variety of indications that map onto distinct populations. In adults, the procedure is used to manage recalcitrant periprosthetic joint infection, failed arthroplasty, femoral neck fracture, septic native hip, and intractable pressure ulcers, predominantly in older, medically debilitated patients.^5–10^ In children, however, GRA is performed largely for painful, dislocated hips in cerebral palsy and related neuromuscular disorders. The pediatric procedure is more recently carried out as a proximal femoral resection interposition arthroplasty, a technically distinct operation that nonetheless shares the same indications.^11–16^

Prior evidence comes from single-institution series confined to narrow age strata, leaving the full age spectrum of the procedure uncharacterized within a single population. Using the TriNetX Research Network, a global federated health research network, we aimed to characterize and compare patient demographics, diagnostic indications, and postoperative complication profiles of patients undergoing GRA, stratified by age group. We hypothesized that pediatric and adult patients undergoing the same coded procedure would differ substantially in their indications and complication profiles.

## Methods

### Study Design and Data Source

This was a retrospective cohort study utilizing the TriNetX Research Network, a global federated health research network that aggregates deidentified electronic health record (EHR) data from participating healthcare organizations (HCOs), primarily in the United States. The TriNetX platform provides access to structured clinical data including demographics, diagnoses coded using the International Classification of Diseases, Tenth Revision, Clinical Modification (ICD-10-CM), procedures coded using Current Procedural Terminology (CPT), medications, and laboratory results. TriNetX complies with the Health Insurance Portability and Accountability Act (HIPAA) and the General Data Protection Regulation (GDPR). All patient data are deidentified in accordance with Section § 164.514(a) of the HIPAA Privacy Rule, as attested by a qualified expert per Section § 164.514(b)(1). Because this study analyzed deidentified data with no direct intervention or interaction with human subjects, institutional review board approval and informed consent were not required. This study adhered to the Strengthening the Reporting of Observational Studies in Epidemiology (STROBE) reporting guideline for observational studies.

### Study Population

Patients who underwent Girdlestone resection arthroplasty (CPT code 27122) between October 1, 2015 and June 8, 2024 were identified using the TriNetX platform. Given this CPT code is specific to the Girdlestone procedure, no exclusion criteria were applied. The study population was stratified into two age-based cohorts: a pediatric cohort (≤ 18 years of age) and an adult cohort (> 18 years of age).

### Epidemiological Characterization

Demographic characteristics including age, sex, race, and ethnicity were collected for each cohort. Age distribution was reported as mean ± standard deviation (SD). To characterize the clinical indications and comorbidity profiles of patients undergoing Girdlestone resection, the ten most prevalent ICD-10-CM diagnosis codes recorded within 3 months of the index procedure were identified and reported for each cohort.

### Outcome Measures

Outcomes of interest were identified using a combination of ICD-10-CM diagnosis codes and CPT procedure codes and included return to the operating room, pain, opioid use, heterotopic ossification, decubitus ulcers, infection (including pyogenic arthritis, osteomyelitis, surgical site infection, and sepsis), and death. Death was ascertained using the TriNetX mortality flag, which integrates data from EHR documentation and external death registries. The specific diagnostic and procedural codes used to define each outcome are detailed in Table 1.

Five-digit numeric codes are CPT (procedure) codes; alphanumeric codes are ICD-10-CM (diagnosis) codes. Italicized entries denote operational criteria applied within the data source rather than a single discrete code.

In M00.x5 and M86.x5, “x” is a wildcard capturing all organism and laterality subcodes for the specified site (hip, thigh).

CPT, Current Procedural Terminology; I&D, incision and drainage; ICD-10-CM, International Classification of Diseases, Tenth Revision, Clinical Modification; OR, operating room; TriNetX, federated electronic health record research network.

### Statistical Analysis

Continuous variables were reported as mean ± SD, since TriNetX does not support median/interquartile range through its standard interface; categorical variables were reported as frequencies and percentages. Outcome risk was calculated as the proportion of patients in each cohort experiencing each outcome event, and between-group comparisons used two-sided Fisher’s exact test, with P values not adjusted for multiple comparisons. Risk ratios and 95% confidence intervals were not available through the platform, so between-group differences are reported as P values only. Because this study was descriptive rather than causal, no adjustment for confounding was performed; potential bias from administrative coding misclassification and under-ascertainment outside the TriNetX network is addressed in the Limitations. Patient-level missing data could not be quantified, as TriNetX reports only network-aggregated counts. All analyses used TriNetX’s built-in statistical tools.

## Results

### Study Population

**Figure 1:**
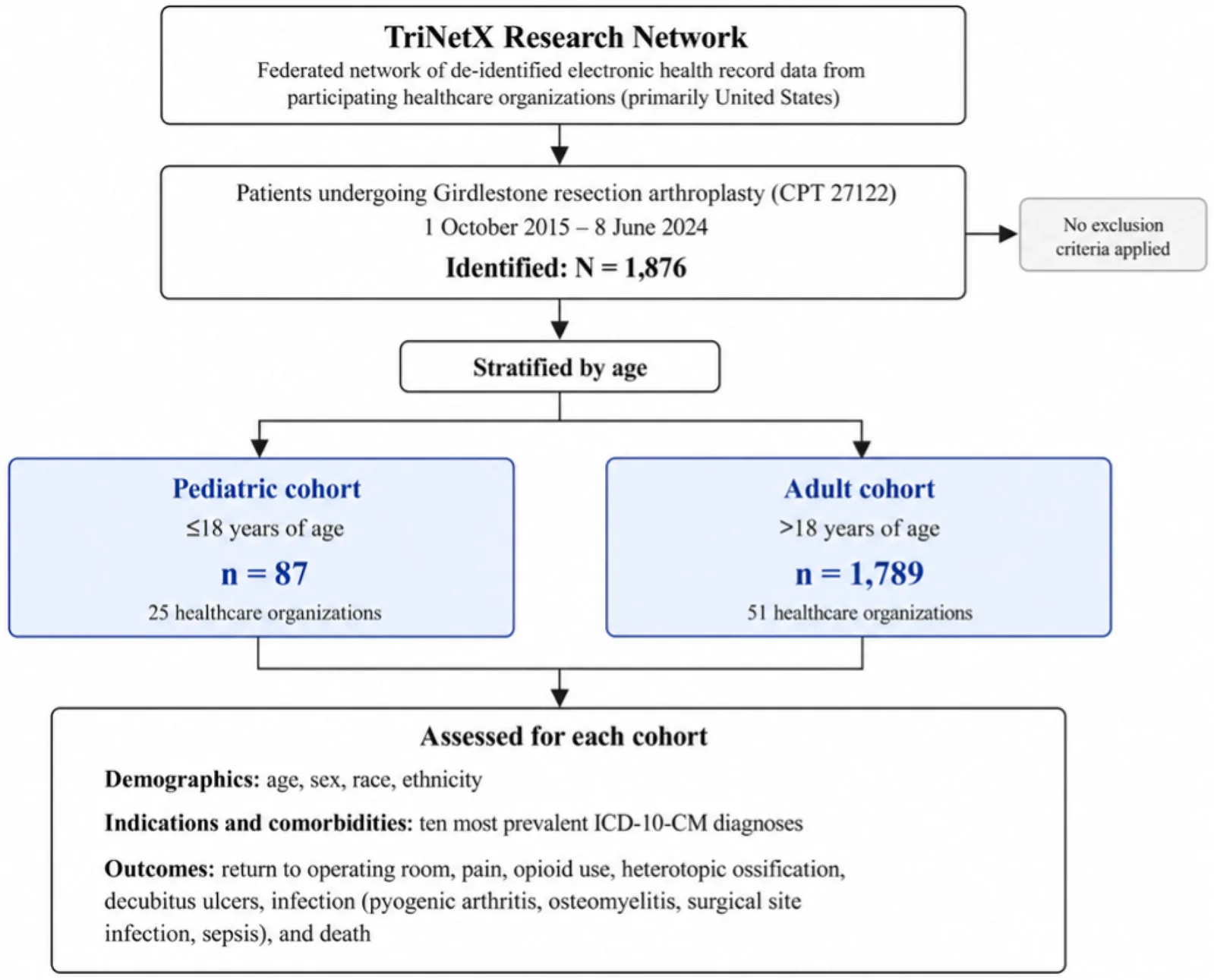
Cohort Selection. Patients undergoing Girdlestone resection arthroplasty (CPT 27122) were identified in the TriNetX Research Network and stratified into pediatric (≤18 year) and adults (>18 years) cohorts.

A total of 1,876 patients who underwent Girdlestone resection arthroplasty were identified (Fig. 1). The pediatric cohort consisted of 87 patients (≤ 18 years) across 25 healthcare organizations, and the adult cohort consisted of 1,789 patients (> 18 years) across 51 health organizations. No patients were excluded.

### Demographics

Mean age was 15 ± 3 years in the pediatric cohort and 58 ± 19 years in the adult cohort. The pediatric cohort was 55.2% male and 44.8% female; the adult cohort was 56.0% male and 44.0% female. By race, the pediatric cohort was 55.2% White, 18.4% Black or African American, 12.6% other race, 11.5% Asian, and 11.5% Native Hawaiian or Other Pacific Islander; the adult cohort was 72.1% White, 18.2% Black or African American, 2.6% other, 1.3% Asian, 0.6% American Indian or Alaska Native, and 0.6% Native Hawaiian or Other Pacific Islander. By ethnicity, the pediatric cohort was 60.9% not Hispanic or Latino, and 33.3% Hispanic or Latino; the adult cohort was 83.8% not Hispanic or Latino, and 6.4% Hispanic or Latino. Percentages for race and ethnicity do not sum to 100% due to exclusion of unknown/unreported status and non-mutually-exclusive race categories in the TriNetX platform.

### Diagnostic Profiles

The ten most frequent diagnoses recorded within three months of the index procedure differed markedly between cohorts (Table 2). The pediatric cohort was characterized by neuromuscular and medically complex conditions, led by cerebral palsy (28%), epilepsy and recurrent seizures (22%), and scoliosis (16%). The adult cohort demonstrated a more heterogeneous profile, led by pain (4%), other joint disorder (4%), pressure ulcer (3%), paraplegia/quadriplegia (3%), and cerebral palsy (3%). The pediatric cohort thus demonstrated a more concentrated diagnostic profile (most frequent diagnosis present in 28% of patients) compared with the adult cohort (most frequent diagnosis in 4%). Cerebral palsy appeared in both cohorts but was far more prevalent among pediatric patients (28% vs 3%).

### Postoperative Outcomes

Pain was common in both groups and did not differ significantly by age (57.5% vs. 60.8%; P = 0.575). All other recorded outcomes occurred exclusively among adults (Table 3). These included decubitus ulcers (28.3%), pyogenic arthritis (23.6%), osteomyelitis (12.8%), death (11.4%), return to the operating room (10.0%), sepsis (9.1%), opioid use (11.7%), surgical site infection (5.3%), and heterotopic ossification (1.1%). All between-group comparisons for these outcomes were statistically significant (P < 0.05), except for heterotopic ossification (P = 1.000).

## Discussion

In this large-scale retrospective cohort study of 1,876 patients, a single procedure code (CPT 27122) identified two clinically distinct populations. Pediatric and adult patients undergoing GRA differed in their associated diagnoses (Table 2) and postoperative complication profiles (Table 3). This divergence is consistent with the way GRA is described across two largely separate bodies of literature: a pediatric neuromuscular salvage operation and an adult salvage operation for infection, failed arthroplasty, and fracture.^3–9,11–16^

The pediatric cohort was defined by neuromuscular and medically complex disease. Cerebral palsy (28%), epilepsy (22%), and scoliosis (16%) were the leading associated diagnoses, followed by dysphagia, respiratory failure, feeding difficulties, and congenital anomalies, a profile consistent with a severely affected, non-ambulatory child. This reflects the population described in prior single-institution studies in which GRA is performed for painful, dislocated hips in cerebral palsy and related neuromuscular disorders.^11–18^ Our data therefore confirm at a national level what previous focused cohorts have long reported.

In contrast, the adult cohort was defined by acquired joint disease, infection, and systemic comorbidities (Table 2). The most frequent associated diagnoses were pain, other joint disorders, pressure ulcer, and paraplegia/quadriplegia. This profile characterizes an older, medically burdened population in which GRA serves as a salvage option for periprosthetic infection, failed reconstruction, fracture, and their sequelae.^5–10,19^ The adult diagnostic and complication profiles demonstrate that the comorbidities leading patients to undergo GRA are the same conditions that drive their postoperative morbidity.

Morbidity was concentrated predominantly in adults (Table 3). Eight of the ten recorded outcomes occurred exclusively in the adult cohort, including decubitus ulcers (28.3%), pyogenic arthritis (23.6%), osteomyelitis (12.8%), death (11.4%), and return to the operating room (10.0%). Each outcome was defined by the diagnostic and procedural codes listed in Table 1. These rates are broadly consistent with the complication and mortality burden reported in adult series.^5,8,9,19^ Most notably, the all-cause mortality of 11.4% is lower than the mortality reported in fracture-specific cohorts, which more likely reflects the broader case mix in our study rather than a truly lower-risk population.^7,8,20^ The coherence between adult indications and adult outcomes is most apparent for pressure ulceration and infection, which appeared as both leading diagnoses (Table 2) and leading complications (Table 3).^6,10,21^ Because diagnoses were ascertained within a window around the index procedure rather than strictly before it, we cannot always distinguish a pressure ulcer or infection that was the indication for surgery from one that followed it, which is a distinction that warrants caution in interpretation.

Pain was the single outcome common to both cohorts and did not differ by age (57.5% vs. 60.8%; Table 3), consistent with the recognized observation that GRA addresses a structural problem while pain often persists.^3,4,22^ Return to the operating room occurred in adults but not in pediatric patients. Although increasing age has been associated with reduced odds of reimplantation after resection for infection, our measure captured any reoperation rather than staged reimplantation specifically and should be interpreted as a related but distinct endpoint.^6,23^

The absence of recorded complications in the pediatric cohort (Table 3) requires careful consideration. It appears to conflict with the pediatric literature, as longitudinal cerebral palsy series consistently report heterotopic ossification, proximal migration, and revision after resection arthroplasty.^13,14,17,18^ However, we identified no such events among our 87 pediatric patients. A plausible explanation is under-ascertainment: administrative coding and a limited follow-up window are unlikely to capture radiographic heterotopic ossification and staged revisions that dedicated imaging-based cohorts detect over years of follow-up. We therefore interpret these zero counts as an absence of recorded events rather than evidence of a low-risk procedure, and we caution explicitly against reading the pediatric results as a demonstration of safety.

Prior studies have been drawn exclusively from single-institution series confined to narrow age strata, either adult or elderly cohorts or pediatric neuromuscular cohorts, leaving the full age spectrum of the procedure uncharacterized within a single population. By describing both cohorts side by side, our findings support an age-specific approach to counseling and risk estimation.

This study has several limitations. First, the retrospective design and reliance on deidentified, coded data preclude causal inference. All findings should be interpreted as associations between age groups and recorded diagnoses or outcomes. Diagnoses and outcomes were ascertained from ICD-10-CM and CPT codes rather than chart review and are therefore subject to coding errors, variable documentation, and the absence of clinical context. Because diagnoses were identified at any time in the record rather than confined to the perioperative period, the diagnostic profiles reported should be regarded as associated comorbidity patterns rather than verified surgical indications. Second, the pediatric cohort was small (n = 87), and the absence of recorded complications in this group should not be interpreted as the absence of risk. With this sample size, an observed rate of zero remains consistent with a true rate of up to approximately 4%. Third, the TriNetX platform reports only the most frequent diagnoses within a cohort and does not support median, interquartile range, or multivariable analysis through its standard interface. Consequently, between-group diagnostic comparisons were limited to diagnoses common to both cohorts. Finally, the network captures care only at participating institutions, so events occurring outside these organizations such as reoperations, complications, or deaths managed elsewhere may be incompletely ascertained, potentially underestimating the true complication and mortality rates.

## Conclusion

Girdlestone resection arthroplasty encompassed two clinically distinct populations: pediatric patients with neuromuscular hip disease and adults with acquired joint disease, infection, and systemic comorbidity. Recorded complications and mortality were concentrated among adults, whereas pain was common in both groups. Age was therefore associated with markedly different indications and outcomes, supporting age-specific counseling rather than a single composite risk estimate for this procedure.

## Figures and Tables

**Table 1:** Operational definitions and corresponding procedure and diagnostic codes used to identify study outcomes.

Outcome	Defining code(s) and criteria
Return to OR	27122 (repeat Girdlestone) 27030 (washout) 10180 (wound I&D) 26992 (drainage of deep abscess, pelvis/hip)
Pain	M25.55 (hip pain) G89.29 (chronic pain) G89.18 (acute postprocedural pain)
Opioid use	F11 (opioid abuse, dependence, use) *Number of prescriptions, quantity per prescription, and tablets*
Heterotopic ossification	M61.0 (myositis ossificans traumatica) M61.9 (calcification and ossification of muscle, unspecified)
Decubitus ulcers	L89 (all stages)
Infection	M00.x5 (pyogenic arthritis, hip) M00.9 (pyogenic arthritis, unspecified) M86.x5 (osteomyelitis, thigh) T81.4 (surgical site infection) A41.9 (sepsis, unspecified organism)
Death	*TriNetX mortality flag*

**Table 2:** Ten most frequent associated diagnoses among patients undergoing Girdlestone resection arthroplasty, stratified by age cohort.

≤ 18 years (n = 87)			≥ 19 years (n = 1789)		
Diagnosis	n	%	Diagnosis	n	%
Cerebral palsy	24	28	Pain, unclassified	68	4
Epilepsy and recurrent seizures	19	22	Other joint disorder, unclassified	63	4
Scoliosis	14	16	Pressure ulcer	61	3
Other functional intestinal disorders	12	14	Essential (primary) hypertension	57	3
Sleep disorders	12	14	Paraplegia/paraparesis and quadriplegia/quadriparesis	57	3
Symptoms/signs concerning food and fluid intake	11	13	Cerebral palsy	55	3
Respiratory failure, unclassified	10	11	Gastro-esophageal reflux disease	46	3
Congenital malformations of trachea and bronchus	10	11	Other disorders of urinary system	44	2
Aphagia and dysphagia	10	11	Other anxiety disorders	44	2
Abnormalities of heart beat	10	11	Other functional intestinal disorders	44	2

Diagnoses are the ten most common within each age cohort, identified by ICD-10 codes. Percentages represent the proportion of patients within the cohort carrying each diagnosis; because patients may have multiple diagnoses, column percentages do not sum to 100%.

**Table 3:** Postoperative outcomes following Girdlestone resection arthroplasty (CPT 27122), stratified by age group.

Outcome	Pediatric (≤ 18 year) n = 87	Adult (> 18 year) n = 1789	*P value* ^a^
Pain	50 (57.5)^a^	1087 (60.8)	0.575
Opioid use	0 (0)	209 (11.7)	**< 0.001**
Return to OR	0 (0)	179 (10.0)	**< 0.001**
Heterotopic ossification	0 (0)	20 (1.1)	**1.000**
Decubitus ulcers	0 (0)	507 (28.3)	**< 0.001**
Surgical site infection (deep or superficial)	0 (0)	94 (5.3)	**0.020**
Pyogenic arthritis	0 (0)	423 (23.6)	**< 0.001**
Osteomyelitis (acute or subacute)	0 (0)	229 (12.8)	**< 0.001**
Sepsis	0 (0)	162 (9.1)	**< 0.001**
Death	0 (0)	204 (11.4)	**< 0.001**

Values are presented as n (%) unless otherwise indicated. Percentages represent the number of patients with the outcome divided by the cohort size, rounded to one decimal place.

a *P* values are from two-sided Fisher’s exact test comparing the pediatric and adult cohorts and are not adjusted for multiple comparisons. Bold values indicate statistical significance (*P* < 0.05).

OR, operating room.

## Data Availability

The data that support the findings of this study were obtained from the TriNetX research network. The data are not publicly available because access is restricted by TriNetX data use agreements and institutional permissions. Researchers with appropriate TriNetX access may reproduce the study using the cohort definitions, diagnosis codes, procedure codes, and outcome definitions described in the Methods section.

## Abbreviations

GRA: Girdlestone resection arthroplasty
ICD-10-CM: International Classification of Diseases, Tenth Revision, Clinical Modification
CPT: Current Procedural Terminology
HIPAA: Health Insurance Portability and Accountability Act
GDPR: General Data Protection Regulation
HCO: healthcare organization
STROBE: Strengthening the Reporting of Observational Studies in Epidemiology
SD: standard deviation
OR: operating room

## References

[1] Girdlestone GR. Acute pyogenic arthritis of the hip: an operation giving free access and effective drainage. 1943. Clin Orthop. 2008;466(2):258–263. 10.1007/s11999-007-0082-6 PMC2505144 18196404

[2] Vincenten CM, Gosens T, van Susante JC, Somford MP. The Girdlestone situation: a historical essay. J Bone Jt Infect. 2019;4(5):203–8. 10.7150/jbji.36618. PMC6831807 31700767

[3] Basu I, Howes M, Jowett C, Levack B. Girdlestones excision arthroplasty: Current update. Int J Surg. 2011;9(4):310–3. 10.1016/j.ijsu.2011.01.012. 21315188

[4] Nester M, Rivera R, Kittur K, Alford N, Huynh TH. Girdlestone resection arthroplasty: indications, outcomes, and complications. Glob J Orthop Res. 2024;4(5). 10.33552/GJOR.2024.04.000593.

[5] Malcolm TL, Gad BV, Elsharkawy KA, Higuera CA. Complication, survival, and reoperation rates following Girdlestone resection arthroplasty. J Arthroplasty. 2015;30(7):1183–6. 10.1016/j.arth.2015.02.011. 25754256

[6] Okewunmi J, Yendluri A, Cordero JK, Patient factors associated with reimplantation after Girdlestone resection arthroplasty for treatment of periprosthetic joint infections of the hip. JAAOS Glob Res Rev. 2024;8(9). 10.5435/JAAOSGlobal-D-24-00005. PMC11404882 39269906

[7] Shah N, Parker MJ. What is the outcome after a Girdlestone resection arthroplasty following a hip fracture? Hip Int. 2023;33(5):948–51. 10.1177/11207000221126135. 36189928

[8] Bellova P, Linne M, Postler AE, Günther KP, Stiehler M, Goronzy J. Girdlestone resection arthroplasty for femoral neck fractures has poorer outcomes than hemiarthroplasty in frail patients with increased risk for arthroplasty-related complications: a retrospective case study of 21 patients. Acta Orthop. 2024;95:61–6. 10.2340/17453674.2024.34901. PMC10826842 38288960

[9] Ghanem D, Covarrubias O, Elsabbagh Z, Poor outcomes of Girdlestone resection arthroplasty in injection drug users: a retrospective study. Antibiotics. 2024;13(8):782. 10.3390/antibiotics13080782. PMC11352147 39200082

[10] Rubayi S, Gabbay J, Kruger E, Ruhge K. The Modified Girdlestone Procedure With Muscle Flap for Management of Pressure Ulcers and Heterotopic Ossification of the Hip Region in Spinal Injury Patients: A 15-Year Review With Long-term Follow-up. Ann Plast Surg. 2016;77(6):645. 10.1097/SAP.0000000000000706. 26808772

[11] Castle ME, Schneider C. Proximal femoral resection-interposition arthroplasty. J Bone Joint Surg Am. 1978;60(8):1051–4. 152765

[12] Baxter MP, D’Astous JL. Proximal femoral resection-interposition arthroplasty: salvage hip surgery for the severely disabled child with cerebral palsy. J Pediatr Orthop. 1986;6(6):681–5. 3793889

[13] Knaus A, Terjesen T. Proximal femoral resection arthroplasty for patients with cerebral palsy and dislocated hips: 20 patients followed for 1–6 years. Acta Orthop. 2009;80(1):32–6. 10.1080/17453670902804935 PMC2823235 19234884

[14] Silverio AL, Nguyen SV, Schlechter JA, Rosenfeld SR. Proximal femur prosthetic interposition arthroplasty for painful dislocated hips in children with cerebral palsy. J Child Orthop. Published online 2016. 10.1007/s11832-016-0775-z PMC5145825 27787761

[15] Chan P, Hsu A, Godfrey J, Outcomes of salvage hip surgery in children with cerebral palsy. J Pediatr Orthop B. 2019;28(4):314–9. 10.1097/BPB.0000000000000566. 30325788

[16] Shaw KA, Hire JM, Cearley DM. Salvage Treatment Options for Painful Hip Dislocations in Nonambulatory Cerebral Palsy Patients. JAAOS - J Am Acad Orthop Surg. 2020;28(9):363. 10.5435/JAAOS-D-19-00349. 31663909

[17] Egermann M. Autologous capping during resection arthroplasty of the hip in patients with cerebral palsy. J Bone Joint Surg Br. 2009;91(8):1007–12. 10.1302/0301-620X.91B8.21808 19651825

[18] Horsch A. Radiological outcomes of femoral head resection in patients with cerebral palsy: a retrospective comparative study of two surgical procedures. Children. 2021;8(12):1105. 10.3390/children8121105 PMC8700049 34943303

[19] Nazemi AK, Upfill-Brown A, Arshi A, Analysis of perioperative outcomes in hip resection arthroplasty. Arch Orthop Trauma Surg. 2022;142(9):2139–46. 10.1007/s00402-021-03833-z. 33625542

[20] Sharma H, Kakar R. Outcome of Girdlestone’s resection arthroplasty following complications of proximal femoral fractures. Acta Orthop Belg. 2006;72(5):555–9. 17152418

[21] Ueno T, Kabata T, Kajino Y, Risk factors for pressure ulcers from the use of a pelvic positioner in hip surgery: a retrospective observational cohort study in 229 patients. Patient Saf Surg. 2020;14:14. 10.1186/s13037-020-00237-7. PMC7137331 32280374

[22] Supnet I, Abiera JE, Alcausin MML, Sumpaico CE. Functional outcomes of an adult with osteogenesis imperfecta after rehabilitation post bilateral Girdlestone procedure. BMJ Case Rep. 2021;14(4):e239884. 10.1136/bcr-2020-239884. PMC8029881 33820804

[23] Ikebe S, Sonohata M, Kitajima M, Kawano S, Mawatari M. Total hip arthroplasty following Girdlestone arthroplasty. J Orthop Sci Off J Jpn Orthop Assoc. 2018;23(3):532–7. 10.1016/j.jos.2018.01.014. 29459080

